# Small RNA-mediated DNA (cytosine-5) methyltransferase 1 inhibition leads to aberrant DNA methylation

**DOI:** 10.1093/nar/gkv518

**Published:** 2015-05-18

**Authors:** Guoqiang Zhang, Pierre-Olivier Estève, Hang Gyeong Chin, Jolyon Terragni, Nan Dai, Ivan R. Corrêa, Sriharsa Pradhan

**Affiliations:** New England Biolabs, Inc., 240 County Road, Ipswich, MA 01938, USA

## Abstract

Mammalian cells contain copious amounts of RNA including both coding and noncoding RNA (ncRNA). Generally the ncRNAs function to regulate gene expression at the transcriptional and post-transcriptional level. Among ncRNA, the long ncRNA and small ncRNA can affect histone modification, DNA methylation targeting and gene silencing. Here we show that endogenous DNA methyltransferase 1 (DNMT1) co-purifies with inhibitory ncRNAs. MicroRNAs (miRNAs) bind directly to DNMT1 with high affinity. The binding of miRNAs, such as miR-155-5p, leads to inhibition of DNMT1 enzyme activity. Exogenous miR-155-5p in cells induces aberrant DNA methylation of the genome, resulting in hypomethylation of low to moderately methylated regions. And small shift of hypermethylation of previously hypomethylated region was also observed. Furthermore, hypomethylation led to activation of genes. Based on these observations, overexpression of miR-155-5p resulted in aberrant DNA methylation by inhibiting DNMT1 activity, resulting in altered gene expression.

## INTRODUCTION

Mammalian epigenetics, including patterns of sequence-specific DNA methylation, are increasingly viewed as predominate drivers in mammalian development and diseases (1). There are three known catalytically active DNA (cytosine-5) methyltransferases, DNMT1, DNMT3A and DNMT3B along with a host of ancillary proteins, including DNMT3L, CFP1 (CXXC finger protein) and UHRF1 (ubiquitin-like containing PHD and RING finger domain protein), that facilitate DNA methylation targeting and maintenance during cell division (2–7). In the classical maintenance DNA methylation model, DNA replication events create hemimethylated and unmethylated CpG dyads in the newly synthesized DNA molecules from fully methylated and unmethylated parental DNA, respectively. Hemimethylated CpG becomes fully methylated by DNMT1, whereas the unmethylated CpG sites remain, thus preserving the site-specific methylation patterns (8,9). DNMT1 is recruited to the replication fork by proliferative cell nuclear antigen (PCNA) and to hemimethylated DNA sites by UHRF1 (6,10–12). These intricate mechanistic events of recruitment and targeting ensure DNMT1 is poised at the site of DNA replication for methyl transfer during cell division. Furthermore, at hemimethylated sites, additional maintenance of methylation fidelity is achieved by catalytic activation of DNMT1 by UHRF1 (13,14).

Recent cumulative genome-wide analysis studies on diverse human cell and tissue types have demonstrated that the majority of CpG sites are stably methylated, and approximately 21% of CpG-methylated sites are dynamically methylated (15). These regions are speculated to play major roles in controlling the transcription networks of cells (15,16). CpGs contained in coding, intronic and extragenic regions generally have higher levels of methylation compared to promoter regions. Both imprinted alleles and inactive X chromosome in mammals are also methylated. Failure to maintain correct methylation patterns often leads to aberrant DNA methylation, promoting human diseases and developmental defects including neurodegenerative, neurological autoimmune diseases and cancers (17). Aberrant DNA methylation often occurs as a result of dysregulation of DNMTs, as reported in various human cancers, including lung, prostate, colorectal and breast cancer (18–20). Although the detailed mechanisms of DNMTs dysregulation are unknown, a number of studies suggest the alteration of DNMT expression, catalysis or targeting may play key roles. Furthermore, long noncoding RNA, microRNA and small RNA are increasingly being identified as potential regulators of DNA methylation (21,22).

Plants and some evolutionary lower eukaryotes such as *Schizosaccharomyces pombe* and *Caenorhabditis elegans* use small interfering RNA (siRNA) to target gene silencing in a sequence-specific manner, resulting in transcriptional repression. However, in the *Arabidopsis* model system, 24-nucleotide small interfering RNAs participate in RNA-directed DNA methylation using Domains Rearranged Methyltransferase 2 (DRM2) (23). Mechanistically, DRM2 was found to exist in complex with the small interfering RNA (siRNA) effector ARGONAUTE4 (AGO4) and preferentially methylate one DNA strand. Therefore, DRM2 is guided to target loci by the AGO4–siRNA complex (24). These transcriptional gene-silencing (TGS) mechanisms account for an estimated 30% of the methylation in *Arabidopsis* genome. Similarly in mammals, a small class of germ-line-specific ncRNA, known as Piwi-interacting RNAs (piRNAs) are shown to guide *de novo* methylation of specific sequences (25). These piRNAs are associated with the Piwi subfamily of the Argonaute protein. Apart from RNA-guided DNA methylation, several families of microRNAs have profound effects on DNA methylation. One such example is the miR-29 family of microRNAs that binds to DNMT3b mRNA and deplete its enzyme levels. Also, downregulation of these microRNAs leads to restoration of the normal pattern of DNA methylation and a decrease in number/size of xenograft mouse tumors (26). Similarly, in proatherosclerotic conditions, reduction of miRNA-152 loosens inhibition of DNMT1, leading to hypermethylation of the ERα gene and a decrease in ERα levels (27). The suggested mechanisms of microRNA-mediated DNA methylation changes are primarily attributed to degradation of DNA methyltransferase mRNAs (28).

Several decades ago, in a classic biochemical study, Bolden *et al*. showed that polyribonucleotides, specifically poly G and homopolymers poly(dC·dG) and poly(dA·dT), are potential inhibitors of partially purified HeLa cell DNA methyltransferase, suggesting this enzyme is regulated *in vivo* by the presence of RNA at specific chromosomal sites, resulting in site-specific DNA methylation (29). Further studies on the potential mechanism of inhibition for DNMT3A proposed a direct binding of RNA molecule on the enzyme (30). Holz-Schietinger and Reich demonstrated a single-stranded RNA molecule that is antisense to the E-cadherin promoter binds tightly to the catalytic domain of DNMT3A and inhibits its activity *in vitro* (30). These observations suggested that a specific RNA may change the epigenetic landscape by selectively targeting DNA methylation machinery. Recently, a new phenomenon of DNA methylation control was discovered, which was mediated by ecRNA (extra-coding RNA) (31). In this study, Di Ruscio *et al*. demonstrated a functional RNA arising from the *CEBPA* locus regulates *CEBPA* methylation. This RNA associates with DNMT1, preventing *CEBPA* gene methylation, which in turn results in robust expression of *CEBPA* mRNA and functional proteins (31). These observations prompted us to dissect the mechanistic events of small RNA-mediated DNMT1 inhibition. We investigated the mechanism of DNMT1 inhibition by small RNA and further focused on a series of microRNAs that can potentially inhibit DNMT1 activity. We further studied the gene expression profile of microRNA-mediated demethylation of the genome.

## MATERIALS AND METHODS

### Methyltransferase assays

Recombinant human DNMT1 was expressed and purified in Sf9 insect cell lines using baculovirus expression system as described before (32). The sequences of RNA oligonucleotides used in this study are listed in Supplementary Table S1. Twenty micromolar of RNA oligonucleotides were resuspended in 10 mM Tris-HCl (pH 8.0) and 100 mM NaCl and incubated at 95°C for 2 min, 65°C for 5 min, 45°C for 10 min, 37°C for 10 min and 25°C for 10 min before use. For formation of G-quadruplex-structured RNA, oligonucleotides were resuspended in a similar buffer, except NaCl was substituted with 100 mM KCl and 2 mM MgCl_2_.

Hemimethylated DNA substrate with the sequence of (C_Me_GG·CCG)_12_ was used for all DNMT1 steady-state biochemistry assays. For determination of the inhibition pattern and *K_i_* of RNAs versus hemimethylated DNA substrate, 20 nM of the DNMT1 enzyme was incubated with various concentrations of hemimethylated DNA substrate in the presence of increasing concentrations of RNA oligonucleotide and 5.328 μM of [^3^H]AdoMet (PerkinElmer). Reactions were performed at 37°C for 30 min as described before (32). Apparent *K_m_* versus RNA concentration was fitted to linear regression, and the *K_i_* was the absolute value of *x* where *y* = 0 in the linear regression function.

To determine the inhibition pattern and *K_i_* of RNAs versus AdoMet, steady-state biochemistry assay was carried out as described earlier, except that hemimethylated DNA was used at 2 μM fixed concentration and varied AdoMet concentrations.

Single-point biochemistry assay was performed by incubating 10 nM of the DNMT1 enzyme with 1 μM of hemimethylatd DNA or 4 ng/μl of poly dI·dC and 5.328 μM of [^3^H]AdoMet in the presence of 5.28 μM of synthetic miRNA oligonucleotide. Reaction was continued for 15 min and [^3^H]CH_3_ incorporation was measured. For bacterial DNA methyltransferase assay, 0.08 U/μl M.SssI, 0.5 U/μl M.HhaI or 0.08 U/μl M.HpaII were incubated with 2 ng/μl of pUC19 DNA and 5.328 μM of [^3^H]AdoMet in the presence of 5 μM synthetic miRNA oligonucleotide. All reactions were performed for 30 min at 37°C in 50 μl final volume.

To study the effect of RNase A on DNA methyltransferase activity, 10 nM of recombinant DNMT1 or immunoprecipitated DNMT1 from HEK293T cells were used. Similar experimental conditions with that of single-point biochemistry assays were used, except that 200 ng/μl RNase A were added to the reaction mixture and incubated for 30 min at 37°C.

### Mapping of the RNA-binding motif in DNMT1

Full-length DNMT1 enzyme was truncated into five fragments and expressed as GST fusions in *Escherichia coli*. Proteins were captured using glutathione sepharose beads (GE healthcare) and washed extensively to get rid of unbound proteins. RNA oligonucleotides were labeled at the 5′ end with [γ-^33^P]ATP (PerkinElmer) using T4 polynucleotide kinase (New England Biolabs), and purified with illustra ProbeQuant G-50 Micro Columns (GE Healthcare). The binding assay was performed in 50 μl reaction volume containing 20 μl of GST-DNMT1 fragment beads, 5 μl of 5 μM labeled RNA oligonucleotides, 5 μl of 10X DNMT1 reaction buffer (New England Biolabs) and 20 μl of RNase-free water. After incubation at 37°C for 10 min, beads were washed three times and resuspended in 200 μl of PBS. The radioactivity bound was measured in liquid scintillator. The amount of RNA oligonucleotide bound to beads was calculated by normalizing the scintillation counts of beads to that of 1 pmol labeled RNA oligonucleotide.

### Cell culture and transfection

HEK293T and HCT116 cells were cultured in DMEM and McCoy's 5A (Gibco) media supplemented with 10% fetal bovine serum, respectively. For genome-wide DNA methylation and transcriptome studies, HCT116 cells were transfected with 2 μM miR-155-5p oligonucleotides or random 23-mer oligonucleotide mixtures for a week using Lipofectamine 2000 (Life Technologies).

### Immunoprecipitation and western blot

HEK293T cells were used for DNMT1–RNA complex immunoprecipitation. Immunoprecipitation was performed as described by Rinn *et al*. (33). Briefly, 10^7^ cells were harvested without cross-linking, washed with PBS and resuspended in 8 ml nuclear isolation buffer (0.32 M sucrose, 10 mM Tris-HCl pH 7.5, 5 mM MgCl_2_ and 1% Triton X-100). After incubation on ice for 20 min, nucleus was pelleted by centrifugation at 2500 x *g* for 15 min at 4°C and resuspended in 1 ml RIP buffer (150 mM KCl, 25 mM Tris-HCl pH 7.5, 5 mM EDTA, 0.5 mM DTT, 0.5% NP-40, 40 U/ml murine RNase inhibitor, 70 μg/ml proteinase inhibitor PMSF). Nuclei were passed through a syringe needle for 40 times and supernatant were collected after centrifugation at 13 000 × *g* for 10 min. Two micrograms of the N-16 goat polyclonal antibody (Santa Cruz) recognizing the N-terminal region of DNMT1 was added to the nuclear extract and incubated at 4°C for 1 h. Then 40 μl of protein G magnetic beads (New England Biolabs) were added and incubated for an additional hour with rotation. After washing three times with RIP buffer, beads were resuspended in 20 μl RIP buffer. Five microliters of DNMT1-bound beads were used for methyltransferase assays. Equal amounts of DNMT1 enzyme in immunoprecipitated samples (for methyltransferase assay) were validated by western blot and DNMT1 monoclonal antibody (ab54759, Abcam).

### Quantitation of DNMT1 binding with miR-155-5p *in vitro*

DNMT1 was immunoprecipitated from HCT116 cells overexpressing the miR-155 pre-miRNA using the DNMT1 antibody mentioned above or normal IgG control. The bound RNA was extracted from immunoprecipitated DNMT1 using Trizol reagent. miR-155-5p was quantified using TaqMan small RNA qPCR assay (Life Technologies).

### Genome-wide DNA methylation analysis using RRBS

One microgram genomic DNA from HCT116 cells transfected with RNAs were digested by MspI, end-repaired, dA-tailed and ligated to methylated loop adaptor using the NEBNext Ultra DNA Library Prep Kit for Illumina (New England Biolabs). After resolving DNA on 2% agarose gels 150–400 bp region was excised and DNA extracted. DNA was bisulfite converted using the EZ DNA Methylation Kit (Zymo Research) and amplified using EpiMark Hot Start Taq DNA Polymerase (New England Biolabs). Biological replicate libraries were constructed and sequenced in separated lanes on the Illumina GAII platform in the 72 bp paired-end reads.

Adaptor sequences were trimmed from RRBS reads using the trim_galore package (http://www.bioinformatics.babraham.ac.uk/projects/trim_galore/) and reads with a Phred score < 20 were discarded. Reads were mapped to hg19 and methylation at CpG sites was extracted using the Bismark package (34). Differential methylation analysis was carried out using the logistic regression statistical test in the methylKit package (35). For calculation of the density CpG in differentially methylated regions, fasta sequences were obtained from hg19 using BEDTools and CpGs were counted (36).

### RNA-seq and transcriptome analysis

RNA-seq libraries were constructed using 1 μg of total RNA as input with the NEBNext Ultra Directional RNA Library Prep Kit (New England Biolabs). Biological triplicate and quadruplicate libraries were made with RNA prepared from control random oligonucleotides or miR-155-5p transfected cells, respectively. Libraries were sequenced on Illumina GAII in separate lanes in 72 bp paired-end reads.

Adaptor and low quality sequences were trimmed and reads mapped to hg19 using the TopHat package (37). Reads mapped to known genes were counted using the HTseq counting tools against the UCSC know genes table (version March 6, 2013) (38). Differential gene expression was analyzed using the DESeq2 package, which fits raw counts to a negative binomial generalized linear model (39). Genes with adjusted *P* value lower than 0.1 (default value in DESeq2) were considered significantly differentially expressed. Gene ontology analysis was carried out using the g:Profiler tools on the biological process and molecular function GO terms (40).

## RESULTS

### RNA binds and specifically inhibits DNMT1

**Figure 1.**
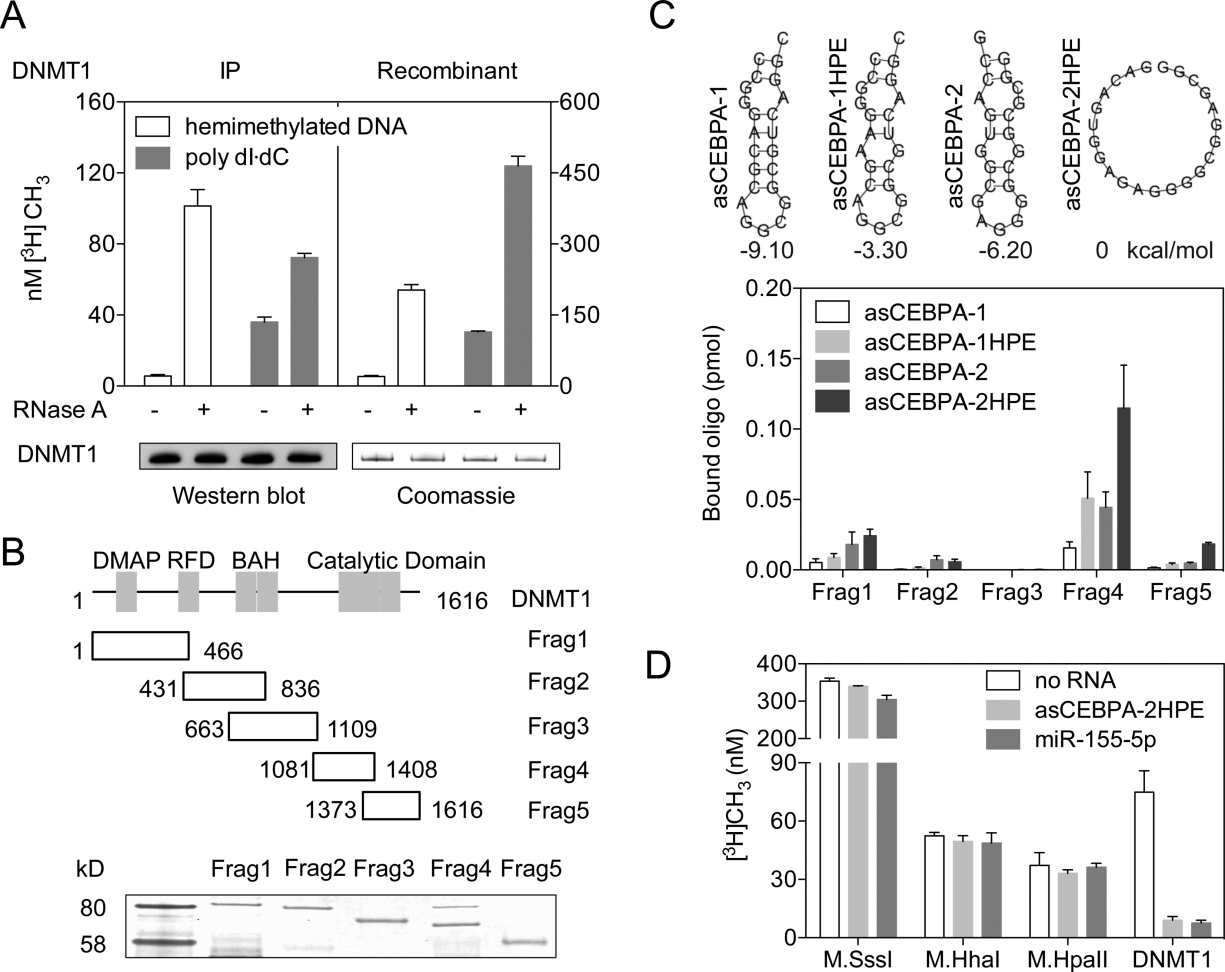
DNMT1 binds to RNA and is inhibited by RNA. (**A**) DNMT1 activity assay with or without RNase A using poly dI·dC or hemimethylated DNA as substrate, left panel DNMT1 immunoprecipitated from HEK293T, and right panel recombinant DNMT1. Lower panel displays respective western blot and Coomassie staining of immunoprecipitated and recombinant DNMT1 after methyltransferase assays. Error bars represent the SD of biological triplicates. (**B**) Schematic diagrams displaying DNMT1 domain structure (upper) and quantitative SDS polyacrylamide gel electrophoresis of GST-DNMT1 fragment fusion proteins used in the RNA-DNMT1 fragment binding assays (Frag (fragment) 1∼5) (lower). DMAP, DNA methyltransferase associated protein binding domain; RFD, replication foci domain; BAH, bromo-adjacent homology domain. (**C**) Secondary structure of RNA oligonucleotides and binding analysis of DNMT1-GST fusions with different oligonucleotides, positional entropy of oligonucleotides was noted beneath the structure diagrams. (**D**) DNA methyltransferase assays in the presence of inhibitory RNA oligonucleotides asCEBPA-2HPE or miR-155-5p using bacterial enzymes (M.SssI, M.HhaI and M.HpaII) or human DNMT1. C and D, Error bars represent the SD of at least three independent experiments each performed in duplicate.

Eukaryotic cells offer a microenvironment of abundant coding and noncoding RNA. To determine if RNA binds and modulates DNMT1 activity, we performed immunoprecipitation of human DNMT1 from the nuclear extract using an antibody against DNMT1. DNMT1 activity was compared on the precipitated samples before and after treatment with RNase A. To our surprise, RNase A treatment increased DNMT1 activity *in vitro*. The methyltransferase activity on hemimethylated DNA was ∼18-fold higher compared to control, whereas 2-fold higher activity was observed on a poly(dI·dC) substrate (Figure 1A). We also carried out the same experiment with baculovirus expressed and purified recombinant DNMT1 (32), where the enzyme was repeatedly washed with 0.5 M salt to get rid of nucleic acid potentially bound in the purification process. Once again, we observed ∼10-fold higher methylation of hemimethylated DNA and about 4-fold higher methylation for poly(dI·dC) substrate (Figure 1A). To exclude the possibility that addition of RNase A may have altered DNMT1 integrity, we probed a portion of the reaction mixture by western blot with anti-DNMT1 antibody for immunoprecipitated samples, or simply resolved the protein by SDS-PAGE and stained for recombinant DNMT1. Indeed, RNase A-treated DNMT1 appeared intact and was of the same molecular mass when compared to control (Figure 1A, lower panel), negating any catalytic activation effort due to proteolysis (41). These results suggest that the inhibitory RNA indeed binds to DNMT1 despite high salt wash during either immunoprecipitation from nuclear extracts or chromatography purifications from Sf9 cells.

It has been previously shown that a functional long ncRNA arising from the *CEBPA* locus, *ecCEBPA*, a long RNA interacts with DNMT1, resulting in prevention of *CEBPA* gene methylation and robust *CEBPA* messenger RNA production (31). RNA oligonucleotides corresponding to the 5′ and 3′ parts of *ecCEBPA* displayed strong affinity for DNMT1 (31). To confirm RNA-binding domain of DNMT1 and its implication in catalysis, we purified five overlapping GST-DNMT1 fusion fragments (Figure 1B). These purified fragments were incubated with two different small RNAs representing the antisense *CEBPA* transcript (asCEBPA-1 and asCEBPA-2) and respective point mutants (asCEBPA-1HPE and asCEBPA-2HPE). These mutants offer higher positional entropy than their corresponding wild-types (Figure 1C, upper panel). Indeed, fragment 4 of DNMT1, which includes a portion of the catalytic domain, bound to all four asCEBPA fragments suggesting that small RNA binds to DNMT1 at the DNA methyltransferase catalytic region (Figure 1C, lower panel).

Since RNA binds to the catalytic domain of DNMT1 and addition of RNase A activates DNMT1, we hypothesized that small RNA or microRNA would be a natural component of DNMT1 enzyme. We immunoprecipitated DNMT1 and observed miR-155-5p enrichment in DNMT1 precipitates, demonstrating a physical interaction between miR-155-5p and DNMT1 (Supplementary Figure S1). To demonstrate the functional role of small RNA-DNMT1 interaction in catalysis, we incubated recombinant purified DNMT1 with asCEBPA-2HPE or miR-155-5p and performed methyltransferase assays on a hemimethylated DNA substrate. Both asCEBPA-2HPE and miR-155-5p inhibited DNMT1 activity (Figure 1D), suggesting RNA as an inhibitor of human DNMT1, presumably by binding to the catalytic domain of the enzyme. To investigate the specificity of RNA-mediated inhibition of DNMT1, three prokaryotic DNA (cytosine-5) methyltransferases with CpG methylation specificity, as that of DNMT1, M.SssI (CG), M.HhaI (GCGC) and M.HpaII (CCGG) were chosen and methyltransferase assays were performed. None of the three prokaryotic DNA methyltransferases showed noticeable inhibition when incubated with asCEBPA-2HPE or miR-155-5p, confirming that the small RNA-mediated inhibition is specific for human DNMT1 (Figure 1D).

### Mechanism of RNA-mediated DNMT1 inhibition

**Figure 2.**
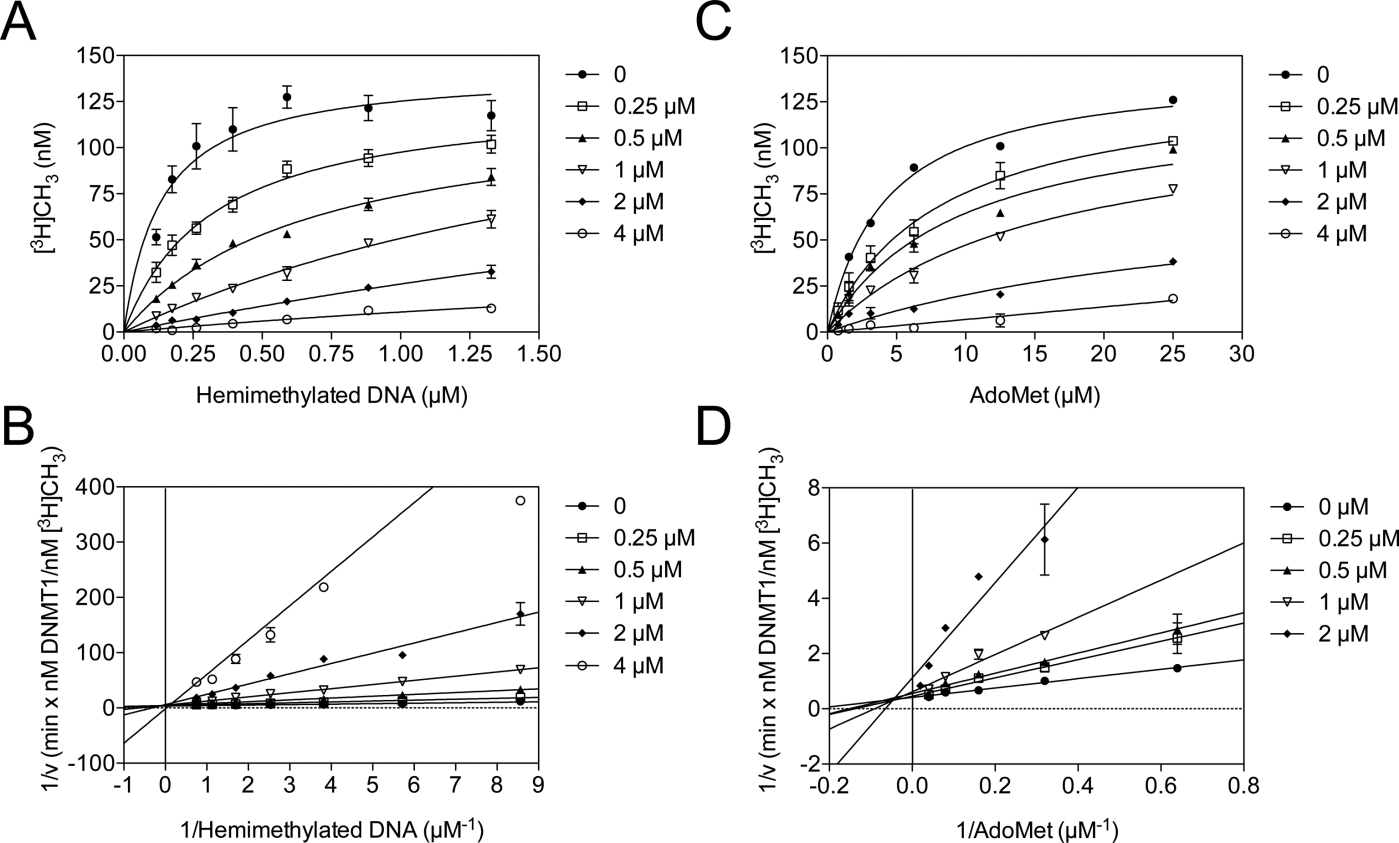
Inhibition patterns of DNMT1 activity by asCEBPA-2 versus hemimethylated DNA and AdoMet substrates. (**A and C**) are representative initial velocity curve of DNMT1 activity in the presence of variable concentrations of asCEBPA-2. The concentrations of [^3^H]CH_3_ incorporated into hemimethylated DNA by DNMT1 were plotted against variable hemimethylated DNA (*A*) or AdoMet concentrations (*C*). (**B and D**), respective double reciprocal plots of (*A*) and (*C*) for fixed asCEBPA-2 concentration. A ∼ D, error bars represent the SD of at least three independent experiments performed in duplicate.

Since certain small RNAs bind and inhibit DNMT1 activity, we performed detailed steady-state enzyme kinetic assays to determine the reaction mechanism. First, we varied the hemimethylated substrate DNA concentration keeping both S-adenosyl-L-methionine (AdoMet) and DNMT1 concentrations fixed and increased the concentration of asCEBPA-2 RNA. As the asCEBPA-2 RNA concentration increased, the apparent *K_m_* value of the enzyme increased without affecting the *V_max_* (Figure 2A), suggesting that asCEBPA-2 RNA-mediated inhibition may be competitive. When we plotted 1/velocity as a function of 1/[hemimethylated DNA] in a double-reciprocal plot, each reaction curve yielded straight lines that intersected on the Y-axis (Figure 2B), confirming that asCEBPA-2 RNA inhibits DNMT1 in a competitive manner, perhaps affecting the hemimethylated substrate binding. Since DNMT1 is a bisubstrate (hemimethylated DNA and AdoMet) enzyme, we further analyzed the effect of asCEBPA-2 on AdoMet substrate binding. We varied the AdoMet concentration keeping both the hemimethylated DNA [S] and DNMT1 [E] concentrations fixed with increasing concentrations of asCEBPA-2 RNA [I]. As the asCEBPA-2 RNA concentration increased, the inhibitor affected the slope and Y-intercepts of the double reciprocal plot (Figure 2C), suggesting asCEBPA-2 RNA-mediated inhibition may be mixed. Re-plots of the 1/velocity as a function of 1/[AdoMet] each yielded a straight line that intersected left of the Y-axis (Figure 2D), confirming asCEBPA-2 RNA inhibits DNMT1 as a mixed inhibitor, perhaps by binding to DNMT1 or to the DNMT1–hemimethylated DNA complex. These results suggest that small RNA binds to DNMT1 directly and alters its hemimethylated substrate binding capacity or by binding to DNMT1–hemimethylated DNA complex. When AdoMet was constant, with varied amounts of hemimethylated DNA as substrate, the *K_i_* was 0.43 μM. Similarly, when hemimethylated DNA was constant with variable AdoMet concentration, the *K_i_* was 0.09 μM (Table 1).

**Table 1.** Inhibition pattern of DNMT1 by small RNAs

Inhibitor RNA	Type of inhibition	*K_i_* (μM)
DNA as variable substrate		
asCEBPA-1	competitive	2.003
asCEBPA-1HPE	competitive	0.918
asCEBPA-2	competitive	0.434
asCEBPA-2HPE	competitive	0.135
miR-9-5p	competitive	0.493
miR-17-5p	competitive	0.317
miR-21-5p	competitive	1.291
miR-92a-1-5p	competitive	0.476
miR-92a-3p	competitive	1.650
miR-127-3p	competitive	0.269
miR-155-5p	competitive	0.028
miR-9-3p	competitive	1.602
miR-16-5p	competitive	0.463
miR-19b-3p	competitive	3.323
miR-20a-5p	competitive	0.561
miR-145-5p	competitive	2.458
miR-146a-5p	competitive	1.031
miR-373-5p	competitive	0.298
AdoMet as variable substrate		
asCEBPA-2	mixed	0.092
miR-17-5p	mixed	0.385

### MicroRNA also inhibits DNMT1

**Figure 3.**
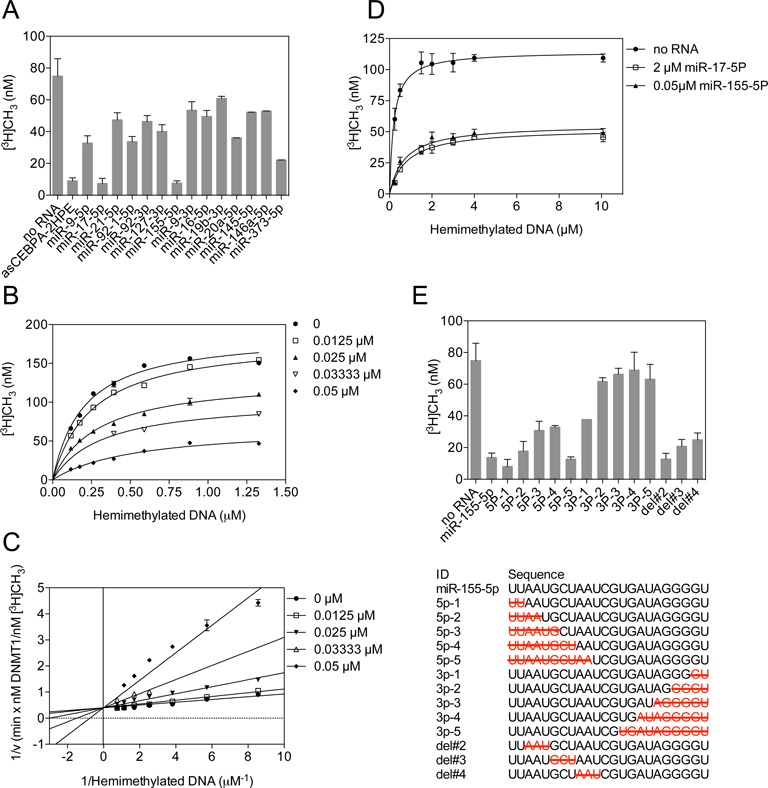
miRNA inhibits DNMT1 activity. (**A**) Single point biochemistry assay showing that miRNAs inhibit DNMT1 activity. (**B**) Velocity curve of [^3^H]CH_3_ incorporation into hemimethylated DNA substrate catalyzed by DNMT1 in the presence of various concentrations of miR-155-5p. (**C**) Double reciprocal plots showing the competitive inhibition pattern of miR-155-5p versus hemimethylated DNA substrate. (**D**) Increasing hemimethylated DNA cannot reverse miRNA-based inhibition. (**E**) Deletion mapping of miR-155-5p (upper) at 5′ or 3′ and internal nucleotides, and bar plot shows the incorporation of [^3^H]CH_3_ in the presence of RNA oligonucleotides (lower). A ∼ E, error bars represent the SD of at least three independent experiments performed in duplicate.

Most cancer cells have aberrant DNA methylation and often overexpress microRNAs. For example, miR-155 and the miR-17∼92 cluster are highly expressed in breast cancer and B-cell lymphomas, respectively (42,43). After surveying the literature, 14 microRNAs were selected to study their effects on DNMT1 activity (Supplementary Table S1). Under identical reaction conditions, almost all microRNAs were able to inhibit DNMT1. However, miR-17-5p, miR-127-3p, miR-155-5p and miR-373-5p showed the greatest inhibition of DNMT1 activity (Figure 3A). We proceeded to investigate if miR-155-5p (one of the best inhibitors, Table 1) mediated inhibition of DNMT1 also follows the same inhibition mechanism as that of asCEBPA-2 RNA. As the miR-155-5p concentration increased, the apparent *K_m_* value of the enzyme increased without affecting the *V_max_*, suggesting miR-155-5p-mediated inhibition may be competitive (Figure 3B). Replots of 1/velocity versus 1/[hemimethylated DNA] for all miR-155-5p concentrations intersected on Y-axis confirming that miR-155-5p also is a competitive inhibitor (Figure 3C). We also performed inhibition profiles for miR-9-5p, miR-17-5p, miR-21-5p, miR-92-1-5p, miR-92-3p, miR-127-3p, miR-9-3p, miR-16-5p, miR-19b-3p, miR20a-5p, miR-145-5p, miR-146a-5p, miR-373-5p and miR-17-5p and observed competitive inhibition in each (Table 1). The inhibition constant *K_i_* varied broadly between 0.03 and 2.4 μM, indicating differential inhibition of DNMT1 by microRNAs, perhaps reflecting their binding affinity and sequence.

We hypothesized that if the binding affinity is a major determinant of microRNA-based inhibition, then the microRNA–DNMT1 complex cannot be dissociated by adding the natural hemimethylated DNA substrate to the reaction mixture. We individually incubated miR-17-5p (*K_i_* = 0.3 μM) and miR-155-5p (*K_i_* = 0.03 μM) with AdoMet and DNMT1. Once the microRNA–DNMT1 complex formed, we added increased amounts of hemimethylated DNA and measured the enzyme activity. Addition of 5-fold excess hemimethylated substrate to a reaction mix containing miR-17-5p (2 μM) only rescued half of normal DNMT1 activity. Similarly, but to a much greater extent, the addition of 200-fold excess hemimethylated substrate to a reaction mix containing miR-155-5p (0.05 μM) also did not rescue DNMT1 activity completely (Figure 3D). These results confirm that microRNA can bind to DNMT1 strongly and inhibit its activity.

We further performed a deletion analysis of miR-155-5p by truncating it from either the 5′ or 3′ end to determine the seed sequence for DNMT1 inhibition (Figure 3E, lower panel). We observed that the first 10 nucleotides from the 5′ end were not essential for DNMT1 inhibition. However, deleting the last two 3′ nucleotides of miR-155-5p relieved ∼50% of the inhibitory effect on DNMT1 and deleting 4–10 3′ nucleotides rescued ∼90% of the activity (Figure 3E, upper panel). This suggests that the 3′ nucleotides of miR-155-5p are essential for inhibition. Indeed, the *K_i_* for the deletion with 5′ 10 nucleotides (5p-5) was 0.12 μM compared to 0.03 μM for the full-length microRNA (Supplementary Figure S2).

The 3′ sequences of miR-155-5p essential for DNMT1 inhibition are guanine-rich sequences tending to form a G-quadruplex structure in solution. We hypothesized that the G-quadruplex form of an RNA molecule may play an active role in the inhibition of DNMT1 activity. Therefore, we chose a telomere RNA sequence and also a short artificial RNA sequence, which we termed SuperG, (Supplementary Table S1) which both have a higher potential of forming G-quadruplex structures in the presence of KCl, and used these to assay inhibition of DNMT1 activity. Indeed, these two small RNA showed greater DNMT1 inhibition when they are annealed in the presence of KCl, compared to control NaCl (Supplementary Figure S3A). The formation of higher molecular weight bands in native polyacrylamide gel electrophoresis (Supplementary Figure S3B) and enhanced absorbance in circular dichroism (CD) spectrum (Supplementary Figure S3C) validated the formation of parallel lattices of G-quadruplexes in DNMT1 assay conditions (44). Taken together, the essential role of the 3′ sequences of miR-155-5p in DNMT1 inhibition, and inhibition of DNMT1 by G-quadruplex structured RNAs suggests that G-quadruplex RNA may fit into the catalytic pocket of DNMT1 and impair the enzyme activity.

### Exogenous miR-155-5p causes aberrant DNA methylation of mammalian genome

To determine the effect of microRNA on genomic methylation in mammalian cell, we transfected mixture of random 23-mer RNA oligonucleotides and miR-155-5p separately into human colorectal cancer cell line HCT116, and compared the DNA methylation in the genome of transfected cells using reduced representation bisulfite sequencing (RRBS) analysis. Libraries were sequenced on an Illumina GAII platform. Of each biological replicate, about 30 million of 72 bp high-quality paired-end reads (Phred score > 20, adaptor trimmed) were mapped uniquely to human genome hg19. With a minimum read coverage threshold of ten, 4 896 172 CpG sites were covered by the four RRBS libraries, which represents about 9% of the total CpG sites in human genome.

**Figure 4.**
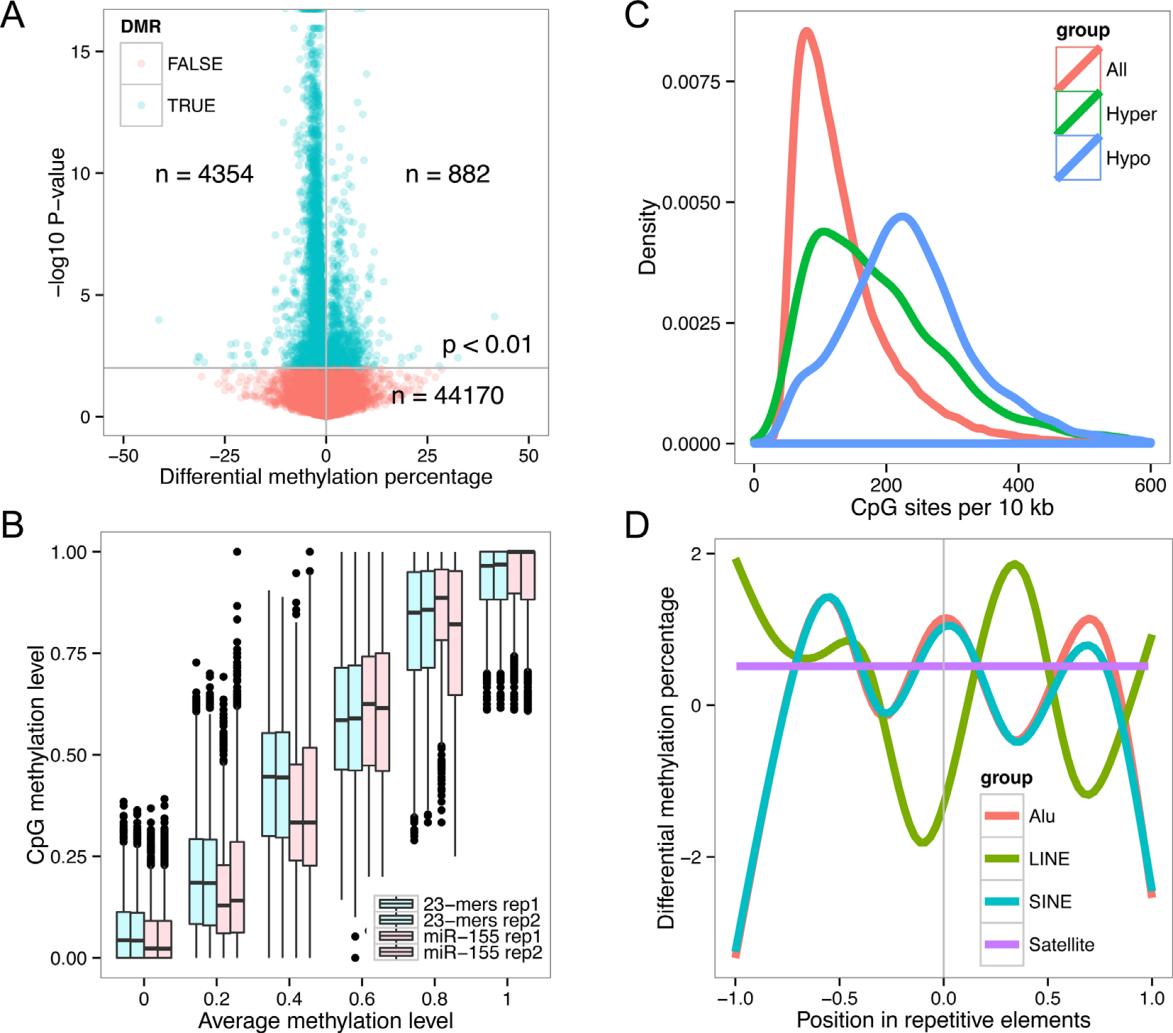
Genome-wide DNA methylation profile of miR-155-5p transfected cells. (**A**) Scatter plot displaying differentially methylated regions in the genome after HCT116 cells were transfected with miR-155-5p or random 23-mers control RNA. The methylation difference (miR-155-5p minus random 23-mers transfected, average value of two biological replicates) was plotted against the –log_10_*P-*value. Green dots represent significantly changed regions. Numbers of differentially methylated regions were annotated in the plot. (**B**) Box plot showing the methylation level in replicates across the bins with increasing methylation level averages. (**C**) CpG site density curve per 10 kb in all regions, hypermethylated regions and hypomethylated regions. (**D**) Average differential methylation in genomic repetitive elements.

Differential methylation analysis was performed on single CpG sites as well as 50 kb and 10 kb tiles. Of the 49 406 nonoverlapped 50 kb genome tiles analyzed, 4354 tiles (8.8% of total tiles) showed hypomethylation in the cells transfected by miR-155-5p compared to the genomic tiles in the cells transfected with random 23-mers with a mean demethylation rate of 2.4%, while 882 tiles (1.8% of total tiles) showed hypermethylation with a mean methylation change rate of 4.0% (logistic regression test, *P* < 0.01). Thus, miR-155-5p causes genome-wide demethylation since the number of hypomethylated tiles is higher than that of hypermethylated tiles (Figure 4A). As expected, the expression level of DNMT1 in the cells transfected with miR-155-5p or random 23-mers is comparable as detected by western blot, RT-qPCR and RNA-seq (Supplementary Figure S4).

At the single base resolution level, 55 225 differentially methylated CpG sites were separated into six bins based on their average methylation level in the four libraries (Figure 4B). Mean methylation levels were significantly lower in the medium-to-low range (bin 0, average demethylation 1.24%, *P* = 0.005, t-test; bin 0.4, average demethylation 6.36%, *P* = 0.007, t-test; other bins are not significantly different) with the most robust demethylation (6.36%) occurring at bin 0.4 (Figure 4B), which represents 10.2% of the total differentially methylated sites. Based on these data, we concluded that miR-155-5p-mediated genome-wide demethylation occurs at the medium-to-low methylated CpG sites.

In another analysis, we calculated the CpG density of nonoverlapping 10 kb differentially methylated tiles. The density of tiles at a particular CpG density was plotted against the corresponding number of CpG sites per 10 kb (Figure 4C). Peaks in the density curve appeared at 78.3, 106.6 and 223.1 CpG sites per 10 kb in the groups containing all the tiles analyzed, significantly hypermethylated or significantly hypomethylated tiles, respectively. This analysis indicated that miR-155-5p-mediated CpG demethylation preferentially occurs in CpG dense regions in the genome. Indeed, by analyzing the distribution of differentially methylated sites, we found that hypomethylated sites are more enriched in CpG islands than the hypermethylated sites, but most of the hypomethylated sites (60.8%) are still located in the non-CpG island regions (Supplementary Figure S5, upper panel). Similarly, hypomethylated sites are enriched to a higher level in promoter regions than hypermethylated sites (Supplementary Figure S5, lower panel). Hence, we conclude that miR-155-5p-mediated genome-wide demethylation events show a preference for medium-to-low level methylated CpG dense regions that may be situated in either CpG islands or elsewhere.

We also examined the methylation of repetitive elements in the genome that is prone to be affected based on DNMTs targeting on the DNA in the cells (45). We analyzed the methylation level of Alu, SINE, LINE and Satellite elements. Differentially methylated CpG sites were assigned to repetitive elements and the average differential methylation level at relative positions of repetitive elements was plotted. Interestingly, the border of SINE and Alu elements and the center of LINE elements showed hypomethylation, while Satellite elements showed hypermethylation (Figure 4D).

### miR-155-5p induced aberrant methylation led to alteration of gene expression

**Figure 5.**
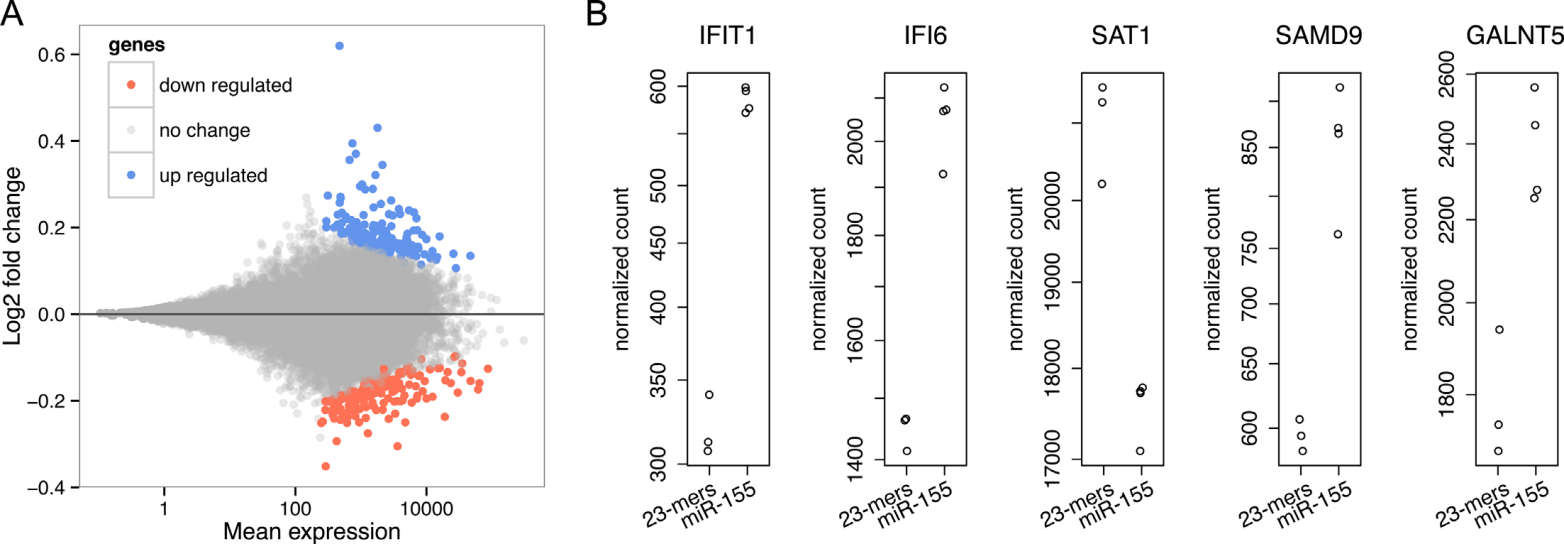
Transcriptome analysis of miR-155-5p and random 23-mers control RNA. (**A**) MA plot analysis of miR-155-5p versus random 23-mers control displaying up- and downregulated genes. (**B**) Normalized read counts of the top five genes with the lowest adjusted *P* values.

**Figure 6.**
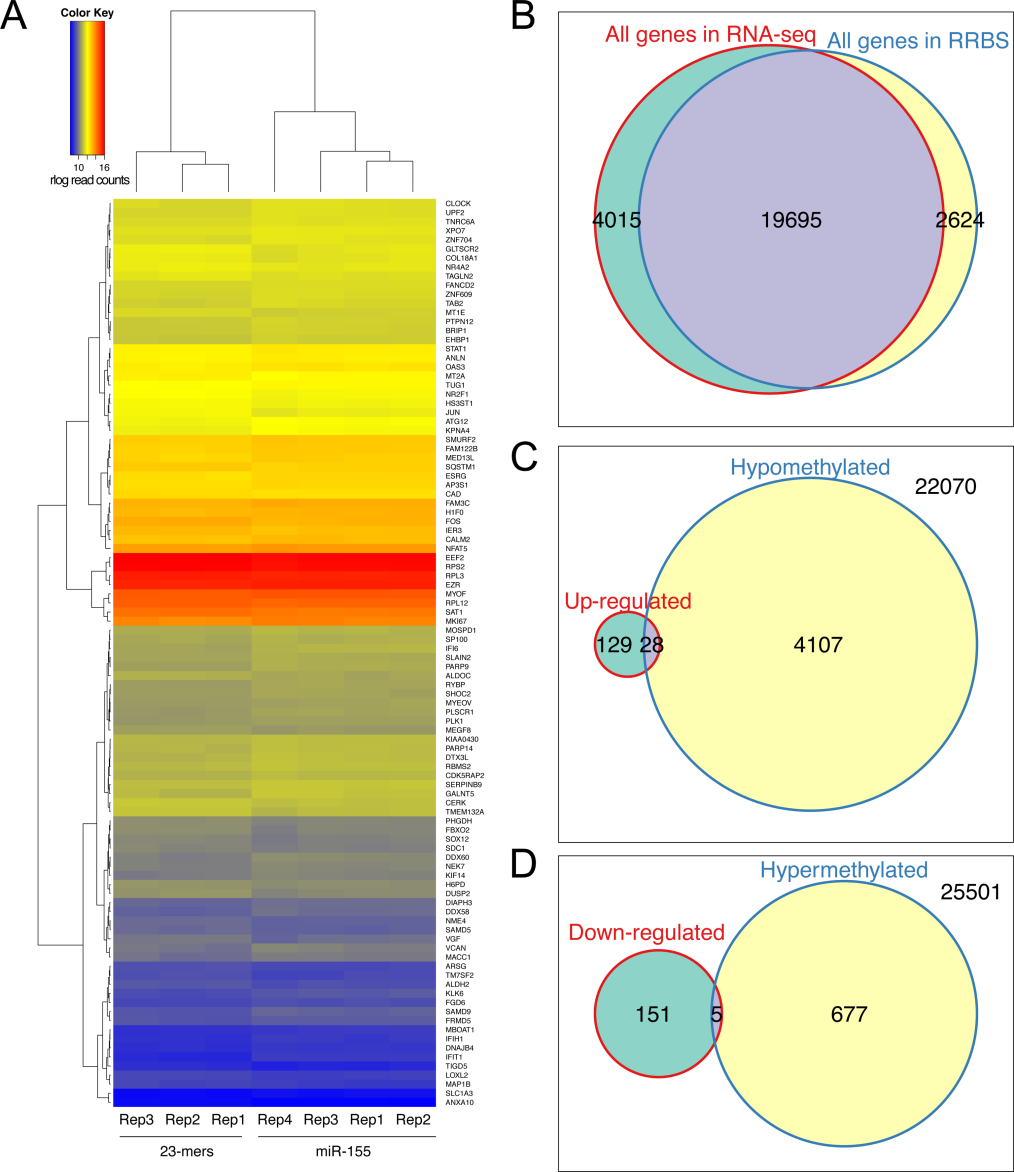
Correlation of differential transcription and differential genomic methylation. (**A**) Heat map of rlog transformed read counts from top 100 genes with lowest adjusted *P* values showing the differential transcription of the random 23-mers control RNA and miR-155-5p transfected cells. Correlation of differential methylation of genome and differential transcription by overlapping the gene list obtained from RRBS and RNA-seq dataset. (**B**) All genes covered by RRBS versus whole transcriptome. (**C**) Upregulated genes versus hypomethylated genes. (**D**) Downregulated genes versus hypermethylated genes.

To determine if microRNA mediated aberrant DNA methylation affects gene expression, we performed a comparative transcriptome analysis of the miR-155-5p and mixture of random 23-mer RNA oligonucleotides transfected HCT116 cells. About 30 million of the 72-bp read pairs were mapped to hg19 for each library, and about 20 million mapped reads in each library could be assigned to a known gene. A total number of 321 genes were found differentially expressed, where 159 genes were upregulated and 162 genes were downregulated in cells transfected with miR-155-5p (Figure 5A). Indeed, by analyzing the top five genes with lowest adjusted *P* value, four of them (*IFIT1*, *IFI6*, *SAMD9* and *GALNT5*) showed elevated expression, while *SAT1* showed reduced expression (Figure 5B). The regularized logarithm (rlog) transformed read counts of the top 100 genes with lowest adjusted *P* value were also plotted on the heatmap demonstrating clustering of random 23-mer RNA oligonucleotides triplicate and miR-155-5p quadruplet displaying differential expression (Figure 6A).

To correlate gene expression change with aberrant DNA methylation by direct inhibition of DNMT1 by miR-155-5p, we performed an overlapping analysis on the gene expression profile and DNA methylation profile (Figure 6B–D). Differential methylation analysis was performed in the context of genes (from -2000 bp upstream of the TSS to the transcription termination site) using the RRBS data, and 4135 genes were found demethylated in the promoter or gene body region while 682 genes were found hypermethylated. By overlapping the hypomethylated gene list with upregulated gene list, 28 hypomethylated genes were found upregulated in the miR-155-5p transfected cells, which represents 17.8% of the total upregulated genes; these genes are *MGEA5, SHOC2, UPF2, IKZF5, MKI67, WAC, CDK1, DNA2, KIF11, MYOF, ARHGAP19, ETS1, ADAR, IQGAP3, EPHA2, MYEOV, KIAA1731, ASPM, KIF14, YOD1, OAS3, MED13L, SH3BP5L, RBMS2, PRIM1, MON2, MDM2* and *IFI6* (Figure 6C). The expression of five hypermethylated genes was found downregulated in the miR-155-5p transfected cells (Figure 6D). Indeed, 19 695 common genes were covered by both RNA-seq and RRBS analysis (at least one CpG site in the promoter and gene body region), which represents 83.1 and 88.2% of the RNA-seq and the RRBS covered genes (Figure 6B). And this indicates that the coverage of RRBS and RNA-seq data across the genome is comparable and the overlapping analysis is not biased. Gene ontology analysis revealed that these differentially expressed genes were significantly enriched in the cell cycle regulation, metabolism and protein binding pathways (Supplementary Figure S6).

## DISCUSSION

Small RNAs are a class of noncoding RNA molecules distributed nonrandomly in the genome. They are frequently located in chromosomal regions susceptible to copy number variation (CNV), associated with cellular functions, gene expression and are often misregulated in cancer. Widespread changes of miRNA expression conferring malignant phenotype development have been reported in multiple cancers (46). Concurrent with alteration of microRNA expression, many cancer cells also overexpress DNA (cytosine-5) methyltransferases facilitating aberrant hypermethylation and silencing of selected tumor suppressor genes (47). This is in contrast with the overall genome-wide hypomethylation in cancer genome, affecting a large number of repetitive elements (48). Several lines of evidence indicate that other mechanisms of gene regulation critically coordinate DNMTs expression, enzyme turnover, catalytic activity and target specificity. Multiple binding sites for transacting factors, presumably involved in post-transcriptional regulation and transcript stabilization leading to modulation of cellular enzyme levels, are found in the 3′ UTR and coding sequence of DNMT mRNA (49). Furthermore, different splice variants of DNMTs have been detected in mammalian cells suggesting these isoforms may have different function and activity.

One example of microRNA targeting DNMT expression is miR-29. In the germ cells of adult rats exposed neonatally to estrogenic analog, EB (estradiol benzoate), and in mouse spermatogonia type B-spermatocyte cell lines transfected with miR-29, decrease of DNMT1, DNMT3a, DNMT3b and anti-apoptotic myeloid cell leukemia sequence 1 (Mcl-1) protein levels was observed (50). The DNMTs decrease was associated with a concomitant increase in transcript levels of previous DNA methylation target genes *L1td1-1ORF1* and *ORF2*, *Cdkn2a* and *Gstp1*, due to hypomethylation. Similarly, in hepatic cell differentiation, miR-148a was critical in direct targeting of DNMT1 expression (51). Other microRNAs, such as miR-152 and miR-17∼92 have also shown transcriptional regulation of DNMT1 (52). Most of these studies have established that an increase or decrease of microRNA expression is correlated with DNMTs mRNA expression.

In our study, we have demonstrated a direct effect of small RNA species, including microRNA, on DNMT1 enzyme activity. These RNAs inhibited DNMT1 activity *via* binding to the catalytic region. Previously, DNMT2, a protein with similar catalytic motif with that of DNMT1 was shown to bind tRNA and confer specificity as tRNA methyltransferase suggesting the catalytic region of DNMTs have evolved as both DNA and RNA binder (53). Once a microRNA binds DNMT1, this complex would lack a turnover mechanism. So, if the binding affinity of RNA and DNMT1 was strong, it would make a catalytically incompetent complex, even in the presence of excess DNA as observed in our study (Figure 3D). We also demonstrated that DNMT1–RNA interaction and inhibition of DNMT1 activity is RNA sequence dependent. Subtle changes in RNA sequence affected the binding of RNA with DNMT1 (Figure 1C). MicroRNA with different sequences exhibited distinct inhibition constant (Table 1). However, we have not described a consensus DNMT1 binding RNA sequence yet. Although, the sequence spectrum of DNMT1 binder RNA may be broad, more sequence analysis of DNMT1 bound small RNA is needed to resolve this issue. Binding and inhibition of DNMT1 by miRNA resulted in aberrant DNA methylation and altered gene expression. Recently, it has been discovered that ecRNA (extra-coding RNA) arising from the *CEBPA* locus regulates *CEBPA* methylation by direct interaction with DNMT1, which results in elevated expression of *CEBPA* mRNA (31). Our mechanistic studies suggest that microRNA, which is a member of the small RNA or short noncoding RNA family (ncRNAs < 200 nucleotides) (54), form complex with DNMT1 and this complex exists in mammalian cells along with DNMT1–ecRNA complex. So, it is possible that there are two distinct complexes of cellular DNMT1, one catalytically active without RNA and the other catalytically inactive with bound RNA. Furthermore, the cellular mechanisms that make catalytically inactive DNMT1 to active is yet to be deciphered.

We also observed a small but significant hypomethylation of the genome in miR-155-5p overexpression cell lines due to inhibition of DNMT1 enzyme activity. The demethylation rate ranged between 0.2 and 36.4% affecting 4135 genes and impacting the expression of a subset of them. We believe demethylation of some of these genes may be inadequate to alter gene expression. Another plausible explanation is that DNA methylation and gene repression may not be strictly correlated, since DNA methylation is not the only epigenetic regulator that affects gene expression. Indeed histone modification marks alone or in combination with DNA methylation could also regulate genes expression (55). In chronic lymphocytic leukemia, positive, negative and nonsignificant correlation between gene expression and DNA methylation were observed for multiple genes (56). Only about 4% of the CpG sites showed a significant correlation between DNA methylation and gene expression (56).

Recently another epigenetic writer, the multi-subunit histone methyltransferase complex, PRC2, is also shown to bind RNA. In a study by Davidovich et al., PRC2 is shown to bind any RNA promiscuously (57). PRC2 was found at transcriptionally active chromatin in its catalytically inactive form. In addition, the authors also find PRC2 bound to repressed genes, and these genes are regulated by catalytically active PRC2-mediated methylation. Thus, the authors propose a model in which promiscuous RNA binding of PRC2 serves as a checkpoint to ensure the PRC2-mediated gene silencing (57). The model indicates that promiscuous RNA inappropriately transcribed from genuine polycomb target gene recruit PRC2, resulting in stimulation of PRC2 activity, H3K27 trimethylation and repression of gene expression (57). Also in another study, PRC2 is guided to its target in an inactive form by RNA and activated by interaction with JARID2 (58). Both studies showed the loading of PRC2 to RNA and the stable recruitment of the complex to chromatin. EZH2 catalytic activity can be highly regulated in independent cellular processes, which are at least in part mediated by the modulation of RNA-binding affinities of EZH2.

It is plausible that a large number of DNA and histone methyltransferases are bound to RNA at the chromatin interface. The lysine methyltransferase SET9 contains a split SET domain and is capable of monomethylating different proteins, and is inhibited by HOTAIR RNA in *in vitro* assays using histone octamers as substrates. Similar observation is also reported for SET8, a histone H4K20 methyltransferase (59). Inactivation of SET9 by RNA would have implication on DNMT1 stability and DNA methylation inheritance, since DNMT1 methylation at K142 by SET9 leads to its degradation by proteosomal pathways (60). Thus it appears that some, if not all-epigenetic writer enzymes may be regulated by RNA. Thus, RNA may be a major gatekeeper for epigenetic inheritance in vertebrates.

## ACCESSION NUMBER

RRBS data and RNA-seq data are available at Gene Expression Omnibus (GSE68027).

## SUPPLEMENTARY DATA

Supplementary Data are available at NAR Online.

SUPPLEMENTARY DATA
