# Supplementary material for: Small RNA-mediated DNA (cytosine-5) methyltransferase 1 inhibition leads to aberrant DNA methylation
Source: Nucleic Acids Res. 2015 May 18;43(12):6112–24. doi: 10.1093/nar/gkv518 (PMC4499142; doi:10.1093/nar/gkv518)
Supplement: SUPPLEMENTARY DATA [file supp_43_12_6112__index.html]

Small RNA-mediated DNA (cytosine-5) methyltransferase 1 inhibition leads to aberrant DNA methylation — Small RNA-mediated DNA (cytosine-5) methyltransferase 1 inhibition leads to aberrant DNA methylation — Small RNA-mediated DNA (cytosine-5) methyltransferase 1 inhibition leads to aberrant DNA methylation — SUPPLEMENTARY DATA 

# Small RNA-mediated DNA (cytosine-5) methyltransferase 1 inhibition leads to aberrant DNA methylation

## SUPPLEMENTARY DATA

- SUPPLEMENTARY DATA
