# Supplementary material for: Small RNA-mediated DNA (cytosine-5) methyltransferase 1 inhibition leads to aberrant DNA methylation
Source: Nucleic Acids Res. 2015 May 18;43(12):6112–24. doi: 10.1093/nar/gkv518 (PMC4499142; doi:10.1093/nar/gkv518)
Supplement: SUPPLEMENTARY DATA [file supp_gkv518_nar-00929-y-2015-File008.pdf]

Small RNA mediated DNA (cytosine-5) methyltransferase 1 inhibition leads to aberrant DNA methylation

Guoqiang Zhang[1], Pierre-Olivier Estève[1], Hang-Gyeong Chin[1], Jolyon Terragni[1], Nan Dai[1], Ivan R. Corrêa Jr.[1], and Sriharsa Pradhan[1]*

[1]New England Biolabs Inc, 240 County Road, Ipswich, MA 01938

*Corresponding author
Ph: 978 380 7227
Fax: 978 921 1350
e-mail: pradhan@neb.com

Supplementary figure legends

Figure S1. DNMT1 binds miR-155 *in vivo*.

DNMT1 was immunoprecipitated from HCT116 cell lines expressing a hairpin form of pre-miR-155 using an antibody recognizing the N-terminal domain of DNMT1 or control normal IgG. Bound miR-155-5p was quantified using TaqMan small RNA qPCR assay. Error bars represent the SD of biological triplicates each performed in duplicate.

Figure S2. Determination of the inhibition pattern and K_i of a miR-155-5p truncate, 5p-5, against hemimethylated DNA.

- A Initial velocity curve of full-length DNMT1 activity in the presence of variable concentrations of 5p-5 (sequence shown in figure 3E).
- B Double reciprocal plots of A for fixed 5p-5 concentration. A and B, error bars represent the SD of at least three independent experiments.

Figure S3. G-quadruplex RNA showed stronger inhibition of DNMT1 activity.

- A DNMT1 activity assay in the presence of G-quadruplex RNA. Error bars represent the SD of at least three independent experiments.
- B Polyacrylamide gel electrophoresis of 60 pmoles of folded telomere RNA in the NaCl or KCl annealing buffer. Higher bands annotated by asterisk in the KCl lane represent G-quadruplex RNA.
- C CD spectrum of telomere RNA annealed in NaCl buffer (purple) or KCl buffer (green).

Figure S4. Expression level of DNMT1 in HCT116 cells transfected with random 23-mers or miR-155-5p.

- A Western blot using HCT116 cells transfected with random 23-mers or miR-155-5p with a mouse monoclonal antibody recognizing DNMT1 (ab54759, Abcam).
- B RT-qPCR detection of DNMT1 transcription. Data were normalized to GAPDH. Error bars represent the SD of biological triplicates.

C rlog transformed counts of the reads that are mapped to the DNMT1 gene in the RNA-seq experiment. Error bars represent the SD of biological triplicates in random 23-mers group and biological quadruplicates in miR-155 group.

Figure S5. Distribution of all, hyper- or hypomethylated CpG sites across CpG island and CpG island shores (upper) or promoter, exon, intron and intergenic (lower) regions.
CpG island shores are defined as the 2000 bp sequences adjacent to CpG islands. Promoter region are defined as the -1000 bp to +1000 bp sequences around the transcription start sites.

Figure S6. GO analysis for differentially expressed genes between the miR-155-5p and random 23-mers control RNA group in transfected cells.

Supplementary table legends

Table S1. MicroRNAs and other small RNA species selected for biochemistry analysis.

Figure S1

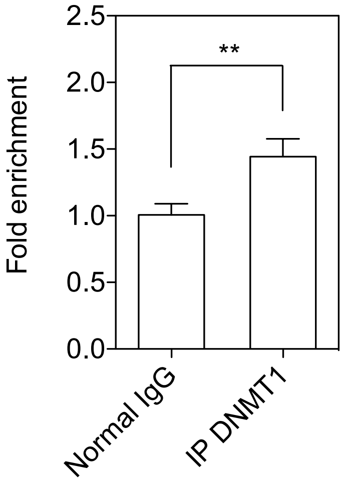

Figure S2

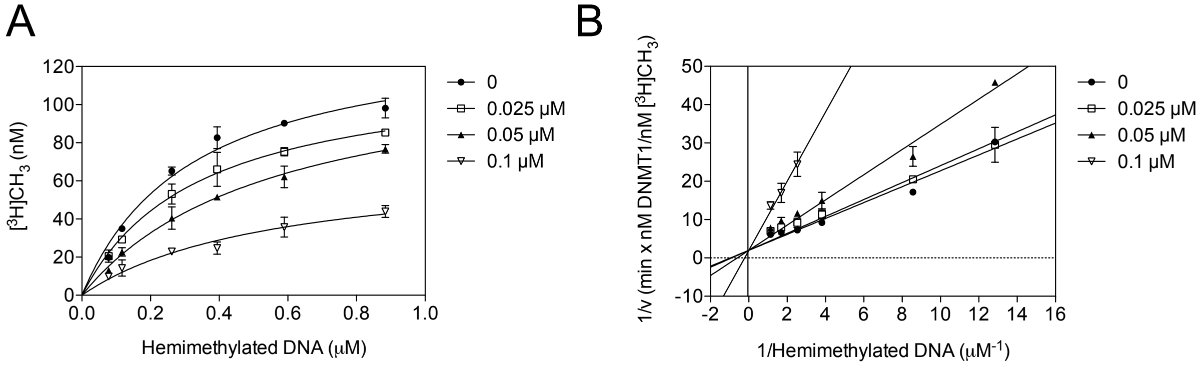

A
[^{3}H]CH$_3$ (nM)
Hemimethylated DNA (μM)
0
0.025 μM
0.05 μM
0.1 μM
B
1/v (min x nM DNMT1/nM [^{3}H]CH$_3$)
1/Hemimethylated DNA (μM^{-1})
0
0.025 μM
0.05 μM
0.1 μM

Figure S3

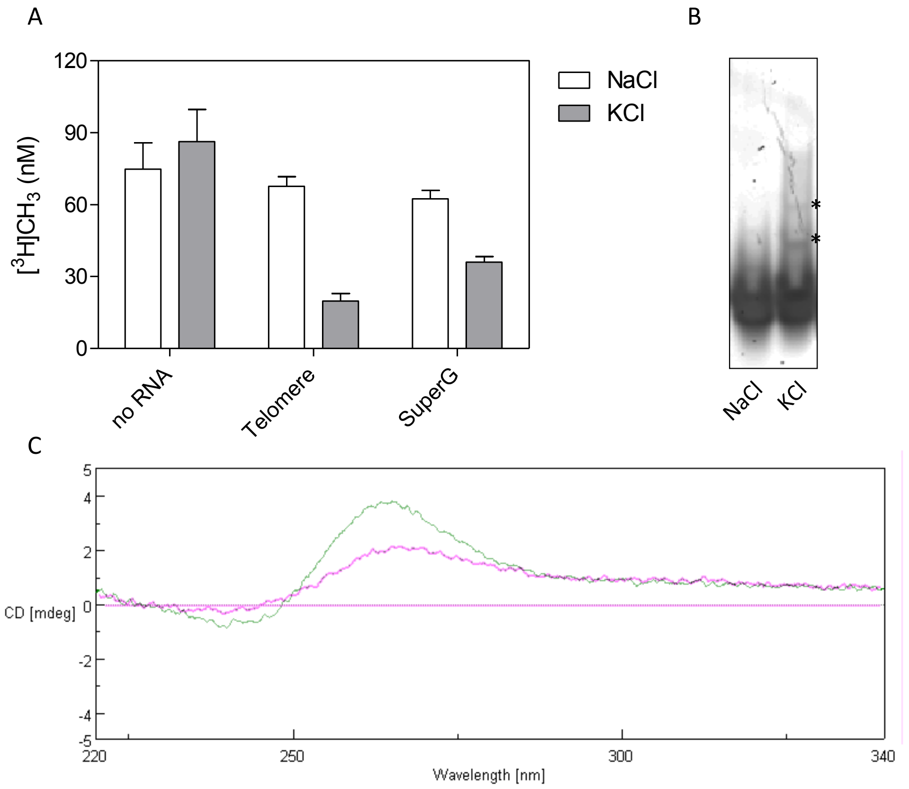
A
120
90
60
30
0
$[^3H]CH_3$ (nM)
NaCl
KCl
no RNA
Telomere
SuperG
B
*
*
NaCl
KCl
C
5
4
2
0
-2
-4
-5
CD [mdeg]
220
250
300
340
Wavelength [nm]

Figure S4

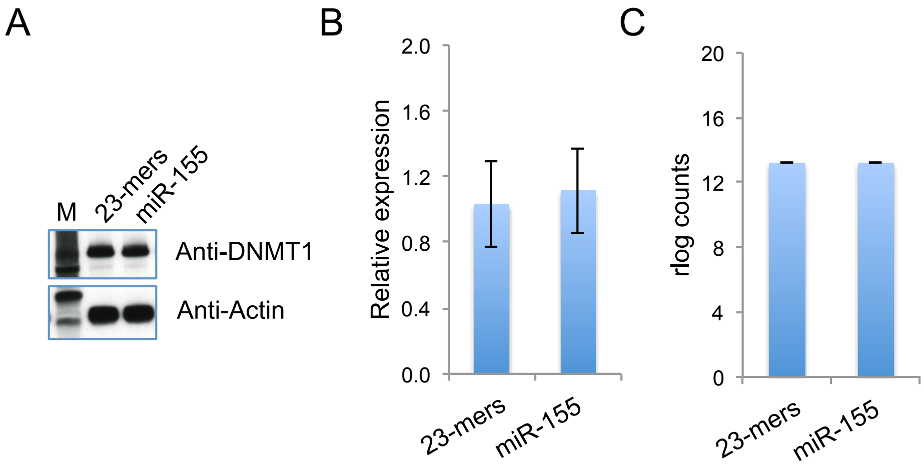
A
M
23-mers
miR-155
Anti-DNMT1
Anti-Actin
B
Relative expression
2.0
1.6
1.2
0.8
0.4
0.0
23-mers
miR-155
C
rlog counts
20
16
12
8
4
0
23-mers
miR-155

Figure S5

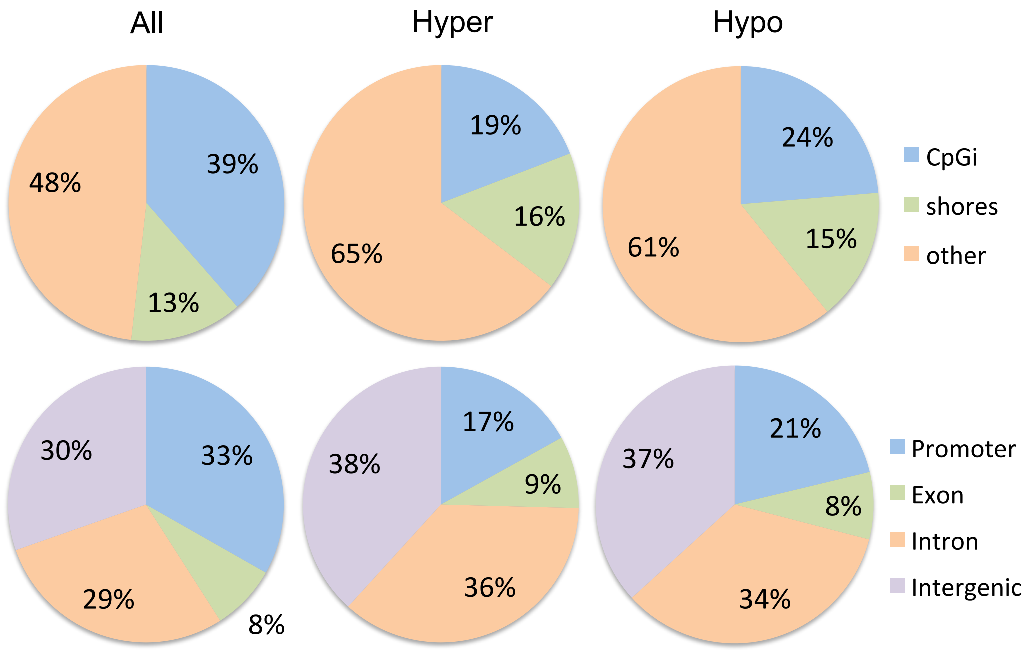

All
Hyper
Hypo
39%
13%
48%
19%
16%
65%
24%
15%
61%
CpGi
shores
other
33%
8%
29%
30%
17%
9%
36%
38%
21%
8%
34%
37%
Promoter
Exon
Intron
Intergenic

Figure S6

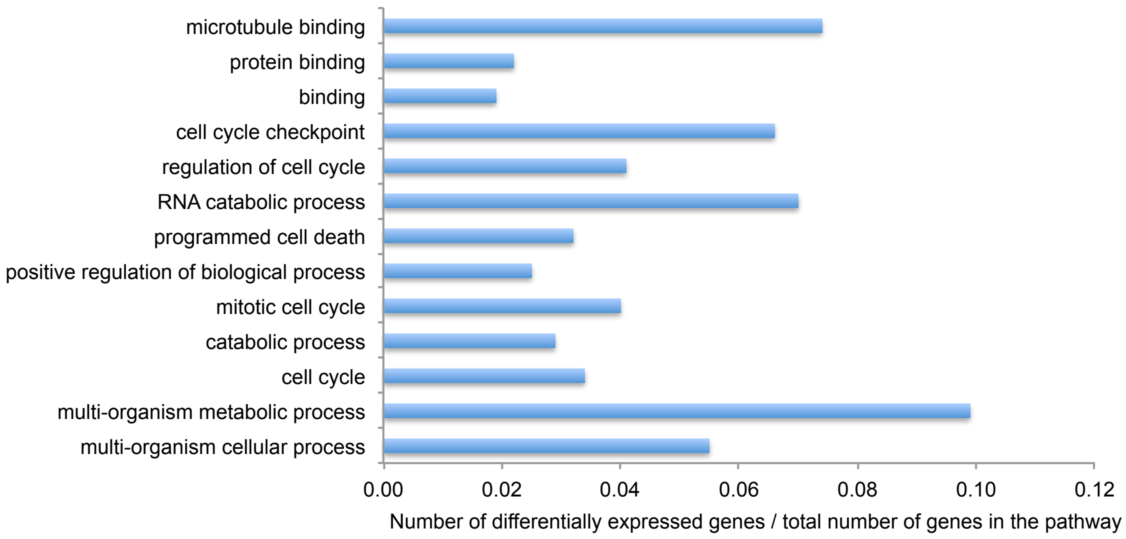

microtubule binding
protein binding
binding
cell cycle checkpoint
regulation of cell cycle
RNA catabolic process
programmed cell death
positive regulation of biological process
mitotic cell cycle
catabolic process
cell cycle
multi-organism metabolic process
multi-organism cellular process
0.00
0.02
0.04
0.06
0.08
0.10
0.12
Number of differentially expressed genes / total number of genes in the pathway

Table S1. MicroRNAs and other small RNA species selected for biochemistry analysis.

Name	Sequences	Involved in cancer types	References
**miR-9-5p**	UCUUUGGUUAUCUAGCUGUAUGA	Breast cancer, gastric adenocarcinoma	(1,2)
**miR-9-3p**	AUAAAGCUAGAUAACCGAAAGU		
**miR-17-5p**	CAAAGUGCUUACAGUGCAGGUAG	Gastric cancer, breast caner, colorectal cancer	(3-5)
**miR-21-5p**	UAGCUUAUCAGACUGAUGUUGA	Multiple types of cancer including breast, ovaries, cervix, colon, prostate cancer and etc	(6-10)
**miR-92a-1-5p**	AGGUUGGGAUCGGUUGCAAUGCU	Colon cancer, breast cancer	(11,12)
**miR-92a-3p**	UAUUGCACUUGUCCCGGCCUGU		
**miR-127-3p**	UCGGAUCCGUCUGAGCUUGGCU	B-cell lymphoma, liver cancer	(13,14)
**miR-155-5p**	UUAAUGCUAAUCGUGAUAGGGGU	Breast cancer, pancreatic cancer	(15-17)
**miR-16-5p**	UAGCAGCACGUAAAUAUUGGCG	Pancreatic cancer, gastric cancer, leukemia, glioma	(18-21)
**miR-19b-3p**	UGUGCAAAUCCAUGCAAAACUGA	Gastric cancer, colon cancer, prostate cancer	(22-24)
**miR-20a-5p**	UAAAGUGCUUAUAGUGCAGGUAG	Gallbladder carcinoma, gastric cancer, cervical cancer	(25-28)
**miR-145-5p**	GUCCAGUUUUCCCAGGAAUCCCU	Lymphoma, renal cell carcinoma, lung adenocarcinoma, colorectal	(29-32)
**miR-146a-5p**	UGAGAACUGAAUUCCAUGGGUU	Thyroid lymphoma, colorectal cancer,	(33,34)
**miR-373-5p**	ACUCAAAAUGGGGGCGCUUUCC	Testicular germ cell tumor, lung cancer, hilar cholangiocarcinoma, breast cancer, hepatocellular carcinoma	(35-39)
**asCEBPA1**	CCGGGACGCAGGCGGCGUCAGGC	Oligonucleotide 1 from asCEBPA transcript	(40)
**asCEBPA1-HPE**	CCGGGAAGCAGGCGGCGUCAGGC	Mutant of oligonucleotide 1 from asCEBPA transcript with higher positional entropy	
**asCEBPA2**	GCCAGUGGCGAGGGGCGGCGCGG	Oligonucleotide 2 from asCEBPA transcript	
**asCEBPA2-HPE**	GACAGUGGAGAGGGGCGGAGCGG	Mutant of oligonucleotide 2 from asCEBPA transcript with higher positional entropy	
**Telomere**	UUAGGGUUAGGG	Telomeric sequence	
**SuperG**	AUGGGGUGGGGU		

References

1. Ma, L., Young, J., Prabhala, H., Pan, E., Mestdagh, P., Muth, D., Teruya-Feldstein, J., Reinhardt, F., Onder, T.T., Valastyan, S. *et al.* (2010) miR-9, a MYC/MYCN-activated microRNA, regulates E-cadherin and cancer metastasis. *Nat. Cell Biol.*, **12**, 247-256.
2. Wan, H.Y., Guo, L.M., Liu, T., Liu, M., Li, X. and Tang, H. (2010) Regulation of the transcription factor NF-kappaB1 by microRNA-9 in human gastric adenocarcinoma. *Mol. Cancer*, **9**, 16.
3. Wang, M., Gu, H., Qian, H., Zhu, W., Zhao, C., Zhang, X., Tao, Y., Zhang, L. and Xu, W. (2013) miR-17-5p/20a are important markers for gastric cancer and murine double minute 2 participates in their functional regulation. *Eur. J. Cancer*, **49**, 2010-2021.
4. Hossain, A., Kuo, M.T. and Saunders, G.F. (2006) Mir-17-5p regulates breast cancer cell proliferation by inhibiting translation of AIB1 mRNA. *Mol. Cell. Biol.*, **26**, 8191-8201.
5. Ma, Y., Zhang, P., Wang, F., Zhang, H., Yang, Y., Shi, C., Xia, Y., Peng, J., Liu, W., Yang, Z. *et al.* (2012) Elevated oncofoetal miR-17-5p expression regulates colorectal cancer progression by repressing its target gene P130. *Nat Commun*, **3**, 1291.
6. Iorio, M.V., Ferracin, M., Liu, C.G., Veronese, A., Spizzo, R., Sabbioni, S., Magri, E., Pedriali, M., Fabbri, M., Campiglio, M. *et al.* (2005) MicroRNA gene expression deregulation in human breast cancer. *Cancer Res.*, **65**, 7065-7070.
7. Iorio, M.V., Visone, R., Di Leva, G., Donati, V., Petrocca, F., Casalini, P., Taccioli, C., Volinia, S., Liu, C.G., Alder, H. *et al.* (2007) MicroRNA signatures in human ovarian cancer. *Cancer Res.*, **67**, 8699-8707.
8. Lui, W.O., Pourmand, N., Patterson, B.K. and Fire, A. (2007) Patterns of known and novel small RNAs in human cervical cancer. *Cancer Res.*, **67**, 6031-6043.
9. Asangani, I.A., Rasheed, S.A., Nikolova, D.A., Leupold, J.H., Colburn, N.H., Post, S. and Allgayer, H. (2008) MicroRNA-21 (miR-21) post-transcriptionally downregulates tumor suppressor Pdcd4 and stimulates invasion, intravasation and metastasis in colorectal cancer. *Oncogene*, **27**, 2128-2136.
10. Folini, M., Gandellini, P., Longoni, N., Profumo, V., Callari, M., Pennati, M., Colecchia, M., Supino, R., Veneroni, S., Salvioni, R. *et al.* (2010) miR-21: an oncomir on strike in prostate cancer. *Mol. Cancer*, **9**, 12.
11. Tsuchida, A., Ohno, S., Wu, W., Borjigin, N., Fujita, K., Aoki, T., Ueda, S., Takanashi, M. and Kuroda, M. (2011) miR-92 is a key oncogenic component of the miR-17-92 cluster in colon cancer. *Cancer Sci.*, **102**, 2264-2271.
12. Nilsson, S., Moller, C., Jirstrom, K., Lee, A., Busch, S., Lamb, R. and Landberg, G. (2012) Downregulation of miR-92a is associated with aggressive breast cancer features and increased tumour macrophage infiltration. *PLoS One*, **7**, e36051.
13. Saito, Y., Liang, G., Egger, G., Friedman, J.M., Chuang, J.C., Coetzee, G.A. and Jones, P.A. (2006) Specific activation of microRNA-127 with downregulation of the proto-oncogene BCL6 by chromatin-modifying drugs in human cancer cells. *Cancer Cell*, **9**, 435-443.

14. Tryndyak, V.P., Ross, S.A., Beland, F.A. and Pogribny, I.P. (2009) Down-regulation of the microRNAs miR-34a, miR-127, and miR-200b in rat liver during hepatocarcinogenesis induced by a methyl-deficient diet. *Mol. Carcinog.*, **48**, 479-487.
15. Zhang, C.M., Zhao, J. and Deng, H.Y. (2013) MiR-155 promotes proliferation of human breast cancer MCF-7 cells through targeting tumor protein 53-induced nuclear protein 1. *J. Biomed. Sci.*, **20**, 79.
16. Gasparini, P., Lovat, F., Fassan, M., Casadei, L., Cascione, L., Jacob, N.K., Carasi, S., Palmieri, D., Costinean, S., Shapiro, C.L. *et al.* (2014) Protective role of miR-155 in breast cancer through RAD51 targeting impairs homologous recombination after irradiation. *Proc. Natl. Acad. Sci. U. S. A.*, **111**, 4536-4541.
17. Que, R., Ding, G., Chen, J. and Cao, L. (2013) Analysis of serum exosomal microRNAs and clinicopathologic features of patients with pancreatic adenocarcinoma. *World J. Surg. Oncol.*, **11**, 219.
18. Gao, L., He, S.B. and Li, D.C. (2014) Effects of miR-16 plus CA19-9 detections on pancreatic cancer diagnostic performance. *Clin. Lab.*, **60**, 73-77.
19. Wang, F., Song, X., Li, X., Xin, J., Wang, S., Yang, W., Wang, J., Wu, K., Chen, X., Liang, J. *et al.* (2013) Noninvasive visualization of microRNA-16 in the chemoresistance of gastric cancer using a dual reporter gene imaging system. *PLoS One*, **8**, e61792.
20. Xi, Y., Li, J., Zan, L., Wang, J., Wang, G. and Ning, Y. (2013) Micro-RNA-16 expression in paraffin-embedded specimen correlates with overall survival of T-lymphoblastic lymphoma/leukemia. *Hum. Pathol.*, **44**, 1011-1016.
21. Li, X., Ling, N., Bai, Y., Dong, W., Hui, G.Z., Liu, D., Zhao, J. and Hu, J. (2013) MiR-16-1 plays a role in reducing migration and invasion of glioma cells. *Anat Rec (Hoboken)*, **296**, 427-432.
22. Wu, Q., Yang, Z., Wang, F., Hu, S., Yang, L., Shi, Y. and Fan, D. (2013) MiR-19b/20a/92a regulates the self-renewal and proliferation of gastric cancer stem cells. *J. Cell Sci.*, **126**, 4220-4229.
23. Kurokawa, K., Tanahashi, T., Iima, T., Yamamoto, Y., Akaike, Y., Nishida, K., Masuda, K., Kuwano, Y., Murakami, Y., Fukushima, M. *et al.* (2012) Role of miR-19b and its target mRNAs in 5-fluorouracil resistance in colon cancer cells. *J. Gastroenterol.*, **47**, 883-895.
24. Tian, L., Fang, Y.X., Xue, J.L. and Chen, J.Z. (2013) Four microRNAs promote prostate cell proliferation with regulation of PTEN and its downstream signals in vitro. *PLoS One*, **8**, e75885.
25. Chang, Y., Liu, C., Yang, J., Liu, G., Feng, F., Tang, J., Hu, L., Li, L., Jiang, F., Chen, C. *et al.* (2013) MiR-20a triggers metastasis of gallbladder carcinoma. *J. Hepatol.*, **59**, 518-527.
26. Li, X., Zhang, Z., Yu, M., Li, L., Du, G., Xiao, W. and Yang, H. (2013) Involvement of miR-20a in promoting gastric cancer progression by targeting early growth response 2 (EGR2). *Int J Mol Sci*, **14**, 16226-16239.
27. Zhao, S., Yao, D., Chen, J. and Ding, N. (2013) Circulating miRNA-20a and miRNA-203 for screening lymph node metastasis in early stage cervical cancer. *Genet Test Mol Biomarkers*, **17**, 631-636.

28. Zhao, S., Yao, D.S., Chen, J.Y. and Ding, N. (2013) Aberrant expression of miR-20a and miR-203 in cervical cancer. *Asian Pac. J. Cancer Prev.*, **14**, 2289-2293.
29. Xia, H., Yamada, S., Aoyama, M., Sato, F., Masaki, A., Ge, Y., Ri, M., Ishida, T., Ueda, R., Utsunomiya, A. *et al.* (2014) Prognostic impact of microRNA-145 down-regulation in adult T-cell leukemia/lymphoma. *Hum. Pathol.*, **45**, 1192-1198.
30. Lu, R., Ji, Z., Li, X., Zhai, Q., Zhao, C., Jiang, Z., Zhang, S., Nie, L. and Yu, Z. (2014) miR-145 functions as tumor suppressor and targets two oncogenes, ANGPT2 and NEDD9, in renal cell carcinoma. *J. Cancer Res. Clin. Oncol.*, **140**, 387-397.
31. Zhao, C., Xu, Y., Zhang, Y., Tan, W., Xue, J., Yang, Z., Zhang, Y., Lu, Y. and Hu, X. (2013) Downregulation of miR-145 contributes to lung adenocarcinoma cell growth to form brain metastases. *Oncol. Rep.*, **30**, 2027-2034.
32. Feng, Y., Zhu, J., Ou, C., Deng, Z., Chen, M., Huang, W. and Li, L. (2014) MicroRNA-145 inhibits tumour growth and metastasis in colorectal cancer by targeting fascin-1. *Br. J. Cancer*, **110**, 2300-2309.
33. Fassina, A., Cappellesso, R., Simonato, F., Siri, M., Ventura, L., Tosato, F., Busund, L.T., Pelizzo, M.R. and Fassan, M. (2014) A 4-MicroRNA signature can discriminate primary lymphomas from anaplastic carcinomas in thyroid cytology smears. *Cancer Cytopathol.*, **122**, 274-281.
34. Hu, X., Li, L., Shang, M., Zhou, J., Song, X., Lu, X., Wang, J., Ying, B. and Wang, L. (2014) Association between microRNA genetic variants and susceptibility to colorectal cancer in Chinese population. *Tumour Biol.*, **35**, 2151-2156.
35. Stelzer, Y., Sagi, I. and Benvenisty, N. (2013) Involvement of parental imprinting in the antisense regulation of onco-miR-372-373. *Nat Commun*, **4**, 2724.
36. Wu, W., He, X., Kong, J. and Ye, B. (2012) Mir-373 affects human lung cancer cells' growth and its E-cadherin expression. *Oncol. Res.*, **20**, 163-170.
37. Chen, Y.J., Luo, J., Yang, G.Y., Yang, K., Wen, S.Q. and Zou, S.Q. (2012) Mutual regulation between microRNA-373 and methyl-CpG-binding domain protein 2 in hilar cholangiocarcinoma. *World J. Gastroenterol.*, **18**, 3849-3861.
38. Keklikoglou, I., Koerner, C., Schmidt, C., Zhang, J.D., Heckmann, D., Shavinskaya, A., Allgayer, H., Guckel, B., Fehm, T., Schneeweiss, A. *et al.* (2012) MicroRNA-520/373 family functions as a tumor suppressor in estrogen receptor negative breast cancer by targeting NF-kappaB and TGF-beta signaling pathways. *Oncogene*, **31**, 4150-4163.
39. Wu, N., Liu, X., Xu, X., Fan, X., Liu, M., Li, X., Zhong, Q. and Tang, H. (2011) MicroRNA-373, a new regulator of protein phosphatase 6, functions as an oncogene in hepatocellular carcinoma. *FEBS J.*, **278**, 2044-2054.
40. Di Ruscio, A., Ebralidze, A.K., Benoukraf, T., Amabile, G., Goff, L.A., Terragni, J., Figueroa, M.E., De Figueiredo Pontes, L.L., Alberich-Jorda, M., Zhang, P. *et al.* (2013) DNMT1-interacting RNAs block gene-specific DNA methylation. *Nature*, **503**, 371-376.
